# PyChelator: a Python-based Colab and web application for metal chelator calculations

**DOI:** 10.1186/s12859-024-05858-8

**Published:** 2024-07-16

**Authors:** Emrulla Spahiu, Esra Kastrati, Mamta Amrute-Nayak

**Affiliations:** 1 Institute of Molecular and Cell Physiology, Hannover Medical School, Carl-Neuberg-Str. 1, 30625 Hannover, Germany; 2 Lassonde School of Engineering, York University, Toronto, M3J 1P3 Canada

**Keywords:** Metal, Chelator, PyChelator, Web application, Python, Calcium, EGTA

## Abstract

**Background:**

Metal ions play vital roles in regulating various biological systems, making it essential to control the concentration of free metal ions in solutions during experimental procedures. Several software applications exist for estimating the concentration of free metals in the presence of chelators, with MaxChelator being the easily accessible choice in this domain. This work aimed at developing a Python version of the software with arbitrary precision calculations, extensive new features, and a user-friendly interface to calculate the free metal ions.

**Results:**

We introduce the open-source PyChelator web application and the Python-based Google Colaboratory notebook, PyChelator Colab. Key features aim to improve the user experience of metal chelator calculations including input in smaller units, selection among stability constants, input of user-defined constants, and convenient download of all results in Excel format. These features were implemented in Python language by employing Google Colab, facilitating the incorporation of the calculator into other Python-based pipelines and inviting the contributions from the community of Python-using scientists for further enhancements. Arbitrary-precision arithmetic was employed by using the built-in Decimal module to obtain the most accurate results and to avoid rounding errors. No notable differences were observed compared to the results obtained from the PyChelator web application. However, comparison of different sources of stability constants showed substantial differences among them.

**Conclusions:**

PyChelator is a user-friendly metal and chelator calculator that provides a platform for further development. It is provided as an interactive web application, freely available for use at https://amrutelab.github.io/PyChelator, and as a Python-based Google Colaboratory notebook at https://colab.research.google.com/github/AmruteLab/PyChelator/blob/main/PyChelator_Colab.ipynb.

**Supplementary Information:**

The online version contains supplementary material available at 10.1186/s12859-024-05858-8.

## Background

The crucial role of metal ions and their ligands, known as chelators, extends across a myriad of biological phenomena, ranging from hemoglobin interacting with iron to chlorophyll binding magnesium. The term “chelate” originating from the Greek word “chela” (the great claw of lobsters [1]), refers to the complex formation between the metal ions and chelators using coordinate bonding.

Maintaining defined free metal ion concentrations in experimental procedures is often critical due to the strong regulatory effects on many isolated protein functions and intracellular systems. Divalent metals like calcium, magnesium, and zinc are commonly encountered in biological studies with chelators like EGTA (ethylene glycol-bis(β-aminoethyl ether)-*N*,*N*,*N*′,*N*′-tetraacetic acid) also known as Egtazic acid, Adenosine triphosphate (ATP), and Ethylenediaminetetraacetic acid (EDTA), which are frequently employed to buffer these metals. However, direct measurement of free metal ions often presents a challenge due to the limited availability of ion-specific electrodes in many laboratories. Consequently, several software calculators have been developed to estimate the free metal concentrations, such as ‘SPECS’ by Fabiato [2], ‘Chelator’ by Schoenmakers et al. [3], ‘Bound and determined’ by Brooks and Storey [4], ‘Calcium’ by Föhr et al. [5], and ‘MaxChelator’ by Bers et al. [6]. The distinguishing factors among these tools lie in their availability, user interface, and the stability constants utilized, with minor variations in calculation methodologies. These calculations take into account the affinity of chelators to metal ions and protons at specific temperature, ionic strength, and pH. As most stability constants are typically measured at standard conditions (e.g., 20 or 25 °C, 0.1 M ionic strength), apparent stability constants are initially computed for the user-defined experimental conditions, followed by the determination of the distribution of species in the solution. Variations in results among different calculators arise from the use of different protonation and metal-affinity stability constants. While older calculators were programmed in languages less accessible today, MaxChelator developed in JavaScript by Chris Patton in 2010 [6] is readily available through modern browsers. However, the numerous calculators present in the literature do not allow the selection of constants from other sources, while some allow only manual editing of the existing constants. Therefore, development of a software that allows easy selection of the available constants and entry of user-defined constants, alongside improvements in user interface and accessibility, is expected to be a valuable advancement.

Metal chelator calculations involve multiple arithmetic operations on numbers with many decimal places, potentially introducing rounding errors, as mentioned for JavaScript-based MaxChelator [6]. JavaScript implicitly converts between strings and floating-point numbers and it relies on the standard double-precision 64-bit binary format, accurate up to 15 digits for integers and 17 for decimals [7, 8]. This inherent limitation in precise representation of the decimals as binary can result in accumulated rounding errors and inaccuracies in computed results. In contrast, Python, a contemporary programming language that achieved high popularity in the scientific community, offers several advantages. Python’s accessibility, extensive scientific libraries and the flexibility in project integration make it an attractive choice [9]. Notably, Python supports a rich variety of numeric data types (integer, float, and complex), and includes a built-in Decimals module, facilitating precise handling of the decimals [10]. User-defined precision in Python yields more reliable results, particularly in scientific computations. Metal-chelator calculators could benefit from such arbitrary-precision arithmetic with the aim to minimize rounding errors.

To address these needs and concerns, we developed PyChelator web application (https://amrutelab.github.io/PyChelator/), an open source program, based on the well-established and widely used Maxchelator framework. PyChelator offers enhanced user experience and customization options. The Python code in a Google Colaboratory notebook makes the PyChelator functionalities readily available to the Python-using scientific community for further development.

## Implementation

PyChelator web application uses JavaScript for calculations and utilizes HTML & CSS for the frontend interface. In Python format, it is accessible as an interactive research notebook (.ipynb) within Google Colaboratory, where users can enter the input parameters via form fields even without coding experience. Upon initiation of the web application, the setup() method is invoked to load the selected chelators and constants, with default constants sourced from the National Institute of Standards and Technology (NIST) Database [11]. A schematic representation of the flow of functions to perform the calculations is depicted in Fig. S1.

## Results and discussion

**Fig. 1:**
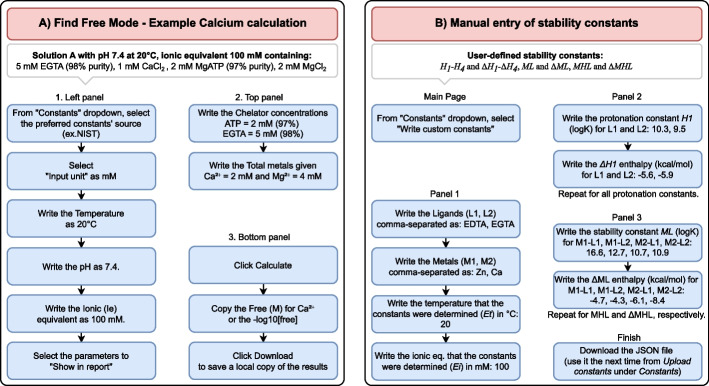
Example uses of PyChelator. **A** An example shows how to calculate the free calcium concentration from the total calcium in the assay buffer used in the in vitro motility assay. **B** The manual entry of stability constants for other metals and chelators. In this case, constants for Zinc and Calcium buffered using EDTA and EGTA were used [11]

The graphical user interface (GUI) of the PyChelator web application is designed for simplicity of use and comprises three panels. In the left panel users select the calculation mode (Free or Total metal concentration), enter the values for different environmental parameters (i.e., temperature, pH, and ionic equivalence), specify the unit for concentration input, and customize the content of the final report. The top panel features the fields for users to input the concentrations of chelators and metals, and the middle panel contains the buttons to do the calculations that are appended one after the other. Finally, a download option enables users to export the results as a single Excel file. An easy-to-follow tutorial of an example calculation and manual entry of constants are included inside PyChelator, and shown in Fig. 1.

## User-friendly input and structured output

**Fig. 2:**
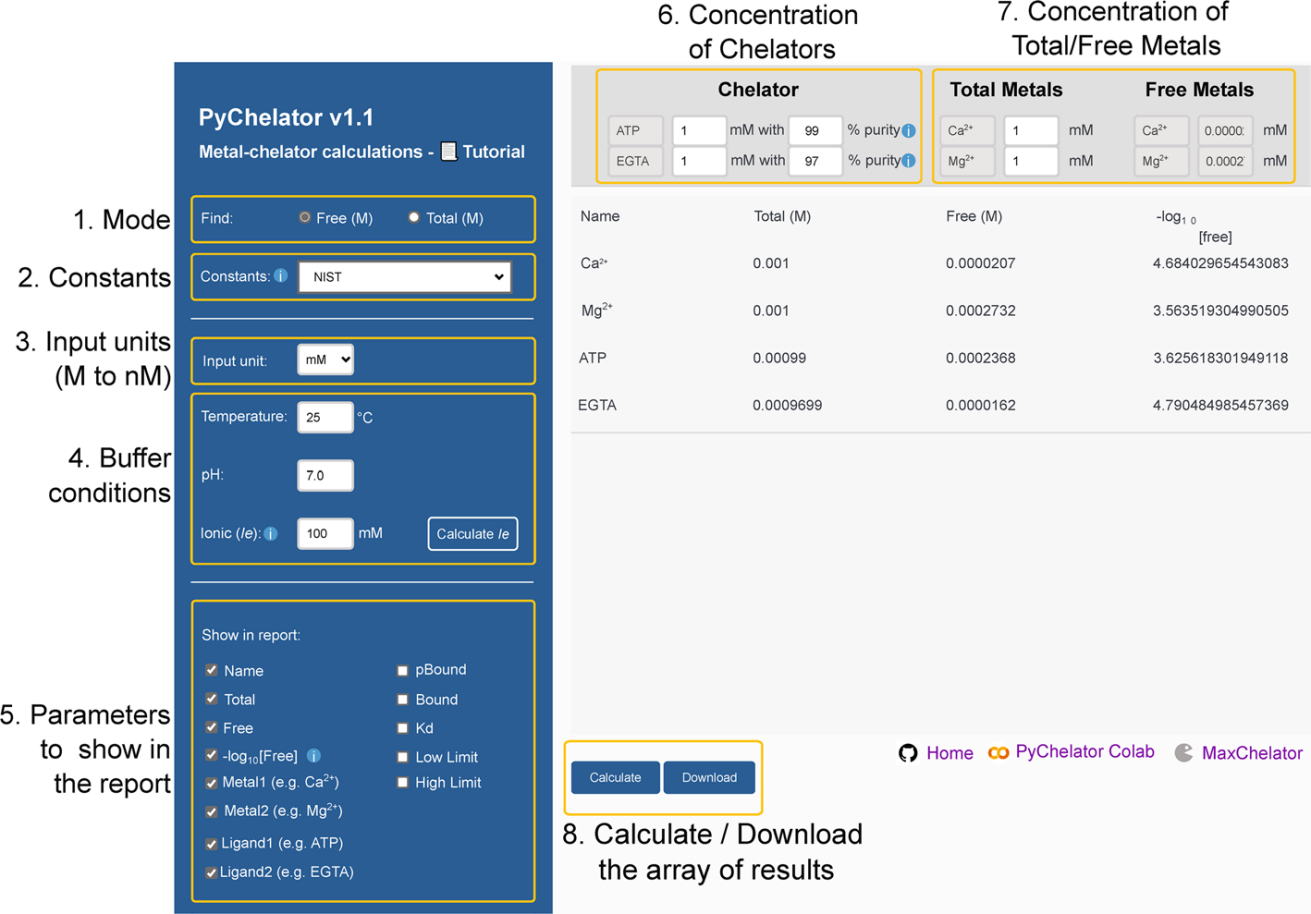
Screenshot of PyChelator graphical user interface (GUI), illustrating the intuitive layout and functionality. The left panel offers the selection of the desired mode of calculation (step 1), followed by the choice of the constants to be utilized (step 2). Four different sources of constants are available. Additionally, user-defined constants can be entered. Users can specify the unit for concentration input of metal and chelators (step 3), the buffer conditions (step 4), and the parameters for inclusion in the final report (step 5). Subsequently, concentration values of chelators together with the measured or supplier-indicated purity should be specified (step 6), followed by the metal concentration (step 7). Finally, results can be calculated through the “Calculate” button and downloaded as a single Excel file (step 8)

PyChelator facilitates the entry of smaller concentrations of chelators and metals through a dropdown menu, enabling entry of values from molar (M) to nanomolar range (M, mM, μM, and nM). Users can utilize the top panel to input the desired values for calculations in the selected unit. Notably, all results are consistently expressed in molar units. Temperature is to be written in degrees Celsius (°C) and ionic strength as equivalence of ions (Ie = 0.5∑Ci|zi|), as explained by Smith and Miller, rather than the standard ionic strength [12]). A function to calculate the ionic equivalence inside PyChelator is also included. Regarding the output, checkboxes were implemented in the “Show in report” section in the left panel, in order to introduce user control over the comprehensive parameters and the metals/ligands to be included in the report. Additionally, PyChelator includes an option for the log-transformed values of the free metal concentrations (− log_10_[free]), corresponding to the pX values (for example pCa for free calcium). In the top panel, a field for purity of chelators was introduced, aiming at addressing the reported significant impact of the chelator purity on calculations [6, 13]. Results are handled in a convenient way. The subsequent calculations are dynamically appended to the middle panel upon clicking the “Calculate” button, and all results can finally be downloaded as a single Excel file. A screenshot of the PyChelator GUI, accompanied by annotations, is presented in Fig. 2. Collectively, these enhancements contribute to an improved user experience for a more efficient and precise use of the calculator, compared to the pre-existing calculators.

## A wide selection and manual input of stability constants

**Fig. 3:**
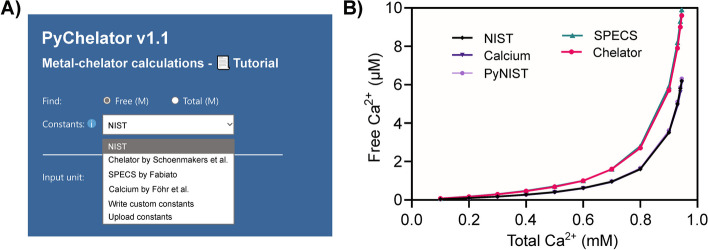
Comparison of PyChelator results obtained from different stability constants. **A** PyChelator offers the selection of stability constants from four sources, and allows the input of user-defined constants. **B** Calculations done in PyChelator using the four sets of stability constants, sourced from the National Institute of Standards and Technology ‘NIST’ [11], ‘SPECS’ by Fabiato [2], ‘Chelator’ by Schoenmakers et al. [3], and ‘Calcium’ by Föhr et al. [5]. Buffers composed of varying total calcium concentration and constant pH of 7.0, temperature of 20 °C, Ionic equivalence (*I*e) of 100 mM, 2 mM ATP, 1 mM EGTA, and 6 mM MgCl_2_. Results represented in a connecting-line plot show that constants sourced from NIST and Calcium software yield similar results to each other and differ from those generated using the constants from SPECS and Chelator program. Notably, the high-precision calculations performed in PyChelator Colab employing the NIST constants and arbitrary precision of 50 decimals in calculations (PyNIST), were similar to those obtained from PyChelator web application using the same constants (NIST)

MaxChelator has two sources of absolute stability constants, i.e., the Chelator program by Schoenmakers et al. [3], and NIST database [11]. The user has to choose the calculator and do the calculations using one of these sources. PyChelator is a single page application where users can switch constants by the use of a dropdown menu. Additionally, there are two new constant sets added. One is sourced from Fabiato and Fabiato [2, 14–16], and the other from the Calcium program by Föhr et al. [5]. The equilibrium constants of ATP were corrected for a temperature of 20 °C, using the standard way by employing the enthalpy of reactions, as explained in Bers et al. [6]. Results obtained using the four sets of stability constants were compared using buffers with varying total calcium concentration, keeping other parameters constant (pH 7.0, temperature of 20 °C, ionic equivalent (*I*e) of 100 mM, 2 mM ATP, 1 mM EGTA, 6 mM MgCl_2_) as shown in Fig. 3. Results obtained using different stability constants show substantial differences. With a narrow range of pX values playing a significant role, as is the case in muscle physiology, where a pCa range of 5–6 is critical in muscle activation, and with results obtained using different constants, it becomes necessary to report the used set of stability constants during such calculations.

PyChelator further enhances user customization by allowing manual input of user-defined stability constants. This feature is accessible under the "Constants" dropdown menu and labeled "Write custom constants". Users can input their data and download it in JSON format for future use. This feature makes it possible to use PyChelator as “Any Metal Any Chelator Calculator”. Some commonly used metal-chelator pairs are included together with references under the “Stability Constants” folder in PyChelator Github repository.

## PyChelator Colab

The aforementioned features of the calculator were composed in a Google Colab Notebook. It is available in the GitHub Repository for download and as a link in the web application. PyChelator Colab offers a cloud-based interactive Python environment that lets users write and execute code in their browsers. Unlike Flask and Django, which are designed to handle server-side operations and require managing server hosting and domain registration (which can be costly), PyChelator Colab and PyChelator web applications do not require server hosting. They can be easily hosted on free platforms like GitHub Pages without the need for managing server hosting and domain registration.

**Fig. 4:**
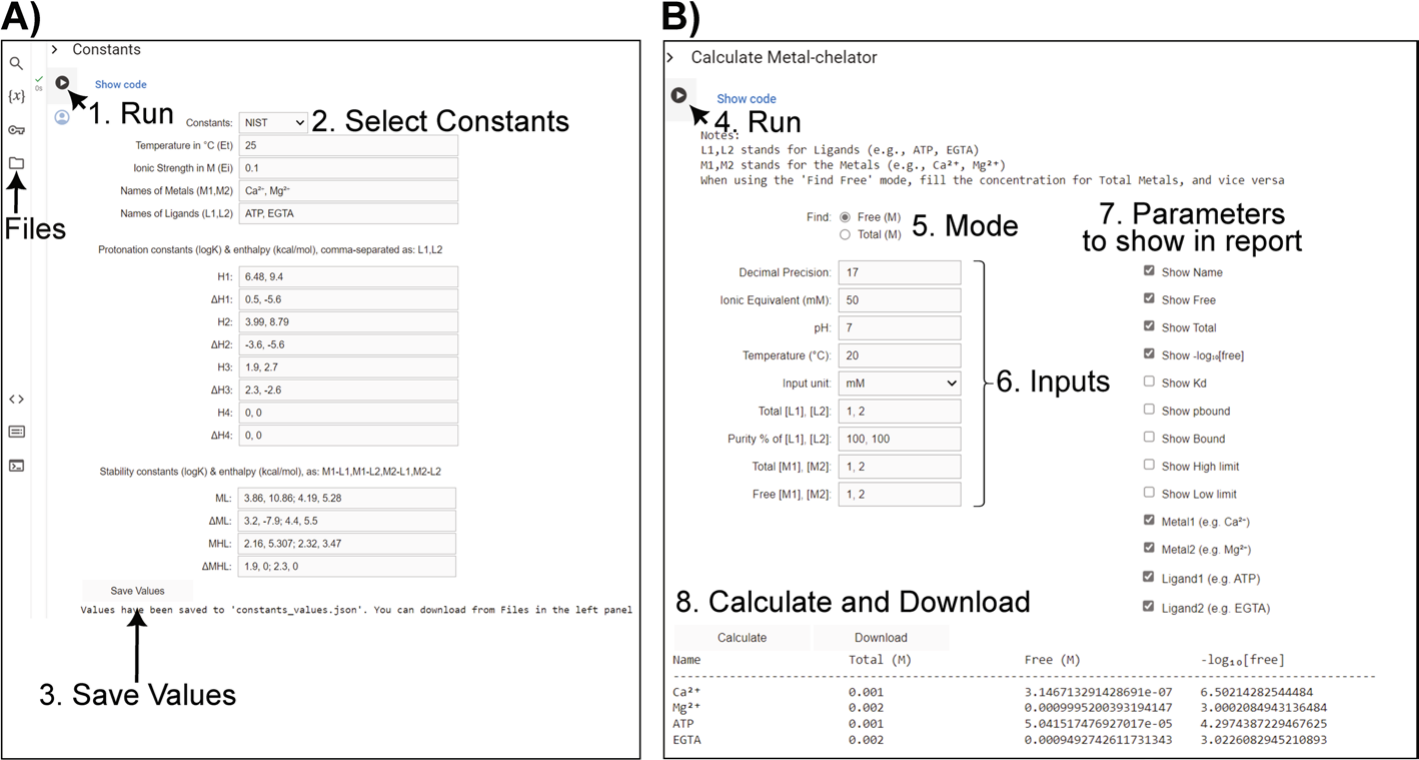
PyChelator Colab GUI. **A** To run a calculation in PyChelator Colab, the user has to run the first code cell "Constants" which enables the dropdown for selection/entry of constants, followed by saving the options through clicking "Save Values". The user can download the constants from "Files" in the left panel. **B** Next, the user has to run the code in the following “Calculate Metal-chelator” section, to input values related to the buffer and to select the output parameters to be included in the report. A field for decimal precision is included to specify the level of precision in decimal places, employing arbitrary precision provided by the Decimals module of Python

The main steps to do the metal chelator calculations in this environment are summarized in Fig. 4. The displayed results can be further employed by experienced users as a part of a pipeline, integrating into a larger project where other data are incorporated. PyChelator Colab is also modified to use the built-in Python Decimal module, introducing user-defined precision in the calculations, which are otherwise limited by double precision in the floating-point arithmetic of Python and JavaScript. The use of arbitrary precision arithmetic gives a higher precision in calculations. Nevertheless, the results obtained from the PyChelator web application, which utilizes JavaScript in calculations, compared to PyChelator Colab, did not show a notable difference (Fig. 3B, NIST vs PyNIST). This implies that any calculations done using MaxChelator algorithms are not limited by precision issues related to the employed JavaScript language. However, although the calculated buffers may offer a good approximation with the measured values, challenges like chelator purity or pH can introduce errors even to the best calculations. Thus, it is always recommended to measure the free metal ions whenever possible [5, 6, 14]. A general comparison of the features offered by PyChelator compared to other calculator software is given in Table 1.

**Table 1:** Comparison of PyChelator to other software

	Programming language	Constants	Input	Output	Refs
PyChelator (2024)	JavaScript, Python	NIST, Schoenmakers, Fabiato, Chelator, User-defined entry	M, mM, μM, nM	Choose parameters, Append, Excel	This work
MaxChelator (2010)	JavaScript	NIST, Schoenmakers, Editable constants file	M	One at a time	[6]
Calcium (1993)	N/A	Martell and Smith, Editable constants	mM	One at a time	[5]
Chelator (1992)	Turbo Pascal	Schoenmakers, Editable constants file	M	One at a time	[3]
SPECS (1988)	Fortran	Fabiato and Fabiato, Editable using WordStar	M	One at a time	[2]

## Limitations

PyChelator validates the input values for temperature (0–40 °C), ionic equivalence (0–500 mM), and pH (0–14). No validation could be introduced to the entry of parameters for the manual entry of constants. PyChelator was prepared to facilitate improvements in these calculations. Future versions may incorporate features such as the possibility to use it for multiple metal-chelator calculations, generation of buffer series with a given composition, calculation of the composition of all complexes in the solution, and downloadable stability constants calculations under different buffer conditions.

## Conclusions

The PyChelator delivers a significant improvement over currently available web applications by offering a user-friendly metal chelator calculator that allows, among others, selection out of four sets of stability constants, input in smaller units, the option to specify user-defined constants, and the convenient ability to download results as a single Excel file. The Python-based PyChelator Colab offers easy customization in the modern programming language with user-defined precision in decimal calculations. Overall, this study not only expands accessibility to metal-chelator calculations but also paves the way for further advancements.

### Supplementary Information

Supplementary Material 1.

## Data Availability

Project name: PyChelator. Project home page: https://amrutelab.github.io/PyChelator/. Source code: https://github.com/AmruteLab/PyChelator. Archived version: 10.5281/zenodo.10674753. Operating system(s): Platform independent. Programming language: Python, JavaScript. Other requirements: None. License: GNU GPL v2. Any restrictions to use by non-academics: E.g., license needed.

## Abbreviations

NIST: National institute of standards and technology
EGTA: Ethylene glycol bis(b-aminoethylether)-N,N,N0,N0-tetraacetic acid
EDTA: Ethylenediaminetetraacetic acid
ATP: Adenosine triphosphate
