# Supplementary material for: PyChelator: a Python-based Colab and web application for metal chelator calculations
Source: BMC Bioinformatics. 2024 Jul 16;25:239. doi: 10.1186/s12859-024-05858-8 (PMC11253343; doi:10.1186/s12859-024-05858-8)
Supplement: Supplementary file 1 — Supplementary Material 1. [file 12859_2024_5858_MOESM1_ESM.pdf]

Supplementary Information

PyChelator: a Python-based Colab and web application for metal chelator calculations

Emrulla Spahiu[1]*, Esra Kastrati[2]*, Mamta Amrute-Nayak[1]

Corresponding Author: Mamta Amrute-Nayak

E-Mail: amrute.mamta@mh-hannover.de

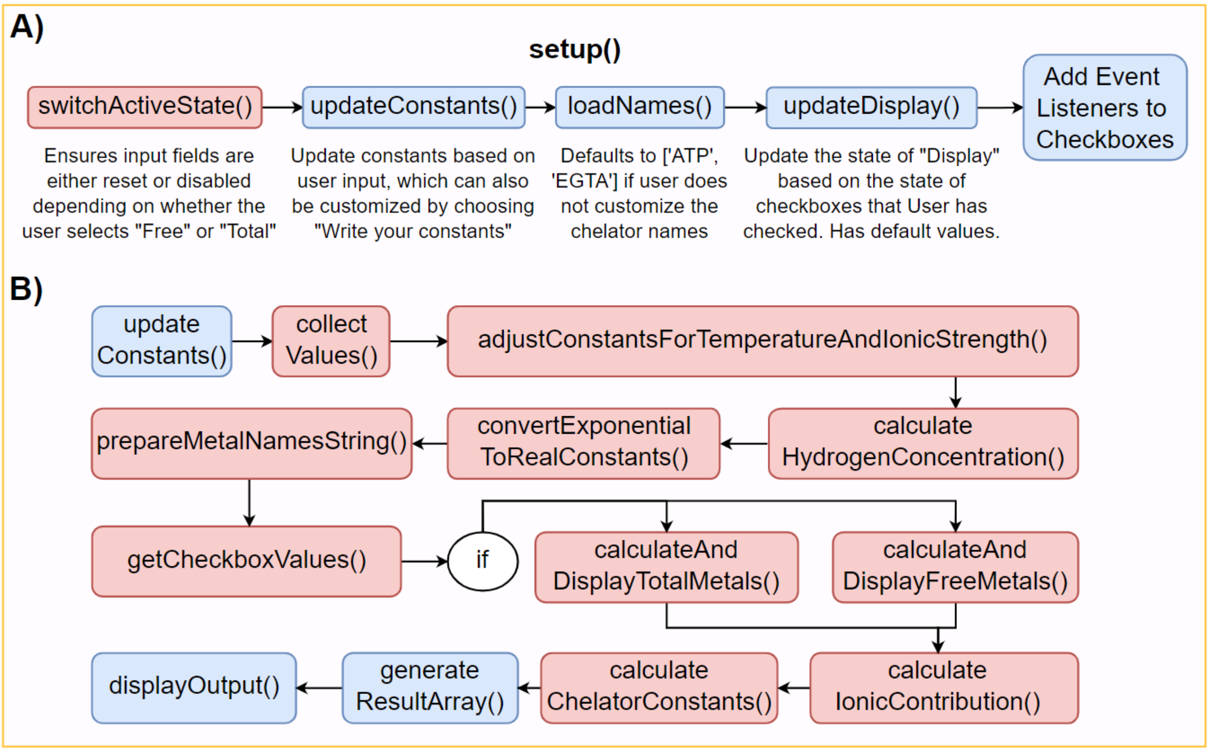

Figure S1. Diagram depicting the functions used in PyChelator. A) Flow of the setup() function. **B)** Sequence of calculations upon calling the docalc() function. Firstly, the function collectValues() retrieves the free or total metal values from the user input, followed by the function adjustConstantsForTemperatureAndIonicStrength(), which adjusts the constants based on the values for temperature and ionic strength (i.e. ionic equivalence) input by the user. Subsequent steps include the calculateHydrogenConcentration() function to calculate the concentration of hydrogen ions in the solution, and the convertExponentialToRealConstants() function to calculate various coefficients and sums based on provided constants and hydrogen ion concentration. Next, the function getCheckboxValues() gets all the checkbox values needed to modify the information outputted to the user. After calculateAndDisplayFreeMetals() or calculateAndDisplayTotalMetals() function is executed, calculateIonicContribution() function calculates the ionic contribution from metal-chelator complexes and free chelators. Finally, the function calculateChelatorConstants() calculates the metal chelation constants and generateResultArray() populates the variable result_array with all the necessary data to generate the file for the user. The function displayOutput() displays the results of the above algorithms. The functions taken from MaxChelator and improved are indicated with red color. The functions indicated with blue were added new. Other new functions that are not depicted in the flowchart are downloadOutput(), which downloads all results as one excel sheet, and calculateionicEquivalent(), which enables calculation and use of ionic equivalence inside PyChelator.

Supplemental information text

Chemical equilibrium of metals and ligands.

The equilibrium between a metal (M) and ligand (L) is represented as:

$$M + L \rightleftharpoons ML \tag{1}$$

Stability constant *K* for the formation of the complex [ML] by the free metal concentration [M] and free ligand concentration [L] is given by the formula:

$$K = \frac{[ML]}{[M][L]} \tag{2}$$

However, ligands (L) such as EGTA do not only bind to divalent metals, but also to hydrogen protons (H). The reactions below express this relationship, as given in the work by Smith and Miller [1]:

$$\begin{array}{ccccc} H_4\text{-}L & \overset{K_4}{\rightleftharpoons} & H_3\text{-}L^{1-} + H^+ & \overset{K_3}{\rightleftharpoons} & H_2\text{-}L^{2-} + H^+ \\ & & & & \upharpoonleft\downharpoonright K_2 \\ M\text{-}H\text{-}L^{1-} & \overset{K_{Ca2}}{\rightleftharpoons} & & & M^{2+} + H\text{-}L^{3-} + H^+ \\ \upharpoonleft\downharpoonright & & & & \upharpoonleft\downharpoonright K_1 \\ H^+ + ML^{2-} & \overset{K_{Ca1}}{\rightleftharpoons} & & & M^{2+} + L^{4-} + H^+ \end{array} \tag{3}$$

K_1 - K_4 are the binding constants of hydrogen to the ligand; K_{Ca1} and K_{Ca2} represent the metal binding constants.

Calcium binds to L^{4-} and HL^{3-} forms of EGTA. If to the EGTA buffer having Calcium, another metal is also added (such as Magnesium), calculations involve solving for a cubic equation (for a sample schematic, see Appendix in [2]). Moreover, if another ligand is added, such as ATP, an iterative calculation must be used to solve for the systems of equations, involving an initial guess, iteration loop and convergence check, until the approximation reaches a certain threshold or the pre-set number of iterations are reached. Before the stability constants can be used in these calculations, they are corrected for ionic strength, temperature and pH. All the calculations are used from the well-established Maxchelator [3, 4]. The flow of the calculations together with the corresponding functions in the code is explained below.

The correction of equilibrium constants for ionic strength and temperature

Temperature correction of equilibrium constants is done based on the Van 't Hoff equation [5, 6]:

$$\log_{10} K' = \log_{10} K + \frac{\Delta H(1/T - 1/T')}{2.303 \times R} \tag{4}$$

where, K' is the corrected equilibrium constant, ΔH is enthalpy of reaction in kcal/mol, T is the temperature in °K, R is the gas constant given by $\mathrm{R} = 1.9872 \times 10^{-3}\ kcal/(mol \cdot {}^\circ K)$.

Ionic strength correction of equilibrium constants is done using the following equation derived from the Debye–Hückel limiting law [1]:

$$\log_{10} K' = \log_{10} K + 2xy\left(\log_{10} f_j - \log_{10} f'_j\right) \tag{5}$$

where, x and y represent the absolute value of charges of the cation and anion in the reaction, the term $\log_{10} f_j$ is the adjustment for activity coefficients at zero ionic strength and $\log_{10} f'_j$ at the desired ionic strength of ion j.

$$\log_{10} f_j = \frac{A \times I_e^{1/2}}{1 + I_e^{1/2}} - b \times I_e \tag{6}$$

$$A = \frac{1.8246 \times 10^6}{(\varepsilon T)^{3/2}} \tag{7}$$

$$\varepsilon = 87.7251 - 0.3974762 \times T + 0.0008253 \times T^2 \tag{8}$$

where, ε is the dielectric constant of water, T is the absolute temperature. The constant A is calculated by substituting the ε and the temperature, and used in the calculation of $\log_{10} f_j$, where b is a coefficient (0.25), and I_e is the ionic equivalent. Ionic equivalent was shown to better explain the empirical relationship of total ionic content to stoichiometric constants [1]. Here, ionic equivalent is used rather than the standard ionic strength, differing in magnitude only in polyvalent ions:

$$I_e = 0.5 \cdot \sum C_i |z_i| \tag{9}$$

where C_i is the concentration and z_i is the charge of the ith ion in the solution.

The corresponding functions in PyChelator:

Web Application (JavaScript): `function adjustConstantsForTemperatureAndIonicStrength()`

PyChelator Colab (Python): `def conadjust(temperature, ionic)`

For Ionic equivalent calculation inside PyChelator:

Web Application (JavaScript): `function ionicEquivalent(concentrations, charges, num_atoms)`

PyChelator Colab (Python): `def ionic_equivalent(concentrations, charges, num_atoms)`

Proton activity coefficient (γ_H) and [H+] calculation

Bers et al. convert pH or $-\log(\text{Hydrogen ion activity})$ into [H+] to be used in Maxchelator calculations [3], using the relationship $10^{-pH} = a_H = \gamma_H[H^+]$, where γ_H is calculated by:

$$\gamma_H = 0.145045 \times e^{(-B \times I_e)} + 0.063546 \times e^{(-43.97704 \times I_e)} + 0.695634 \tag{10}$$

The constant B is defined as $B = 0.522932 \times e^{(0.0327016 \times T)} + 4.015942$, where T is temperature in °C.

The corresponding functions in PyChelator:

Web Application (JavaScript): `function calculateHydrogenConcentration()`

PyChelator Colab (Python): `def calcH()`

Conversion of constants from exponentials to real values

The corresponding functions in PyChelator:

Web Application (JavaScript): `function convertExponentialToRealConstants()`

PyChelator Colab (Python): `def makekon()`

Calculation of apparent Ca^{2+} affinity constant (K'_{Ca})

The apparent affinity of Calcium is calculated in Maxchelator by:

$$K'_{Ca} = \frac{K_{Ca}}{1+[H^+]K_{H1}+[H^+]^2K_{H1}K_{H2}+[H^+]^3K_{H1}K_{H2}K_{H3}+[H^+]^4K_{H1}K_{H2}K_{H3}K_{H4}} + \frac{K_{Ca2}}{\frac{1}{[H^+]K_{H1}}+1+[H^+]K_{H2}+[H^+]^2K_{H2}K_{H3}+[H^+]^3K_{H2}K_{H3}K_{H4}} \quad (11)$$

where, K_{H1} to K_{H4} are the acid association constants for the chelator, K_{Ca} and K_{Ca2} are the Ca^{2+} association constants to the L^{4-} and HL^{3-} forms of the ligand [6].

The corresponding functions in PyChelator:

Web Application (JavaScript): `function calculateChelatorConstants()`

PyChelator Colab (Python): `def makekd()`

References:

1. Smith GL, Miller DJ. Potentiometric measurements of stoichiometric and apparent affinity constants of EGTA for protons and divalent ions including calcium. Biochimica et Biophysica Acta (BBA) - General Subjects. 1985;839:287–99.

2. Chang D, Hsieh PS, Dawson DC. Calcium: A program in basic for calculating the composition of solutions with specified free concentrations of calcium, magnesium and other divalent cations. Computers in Biology and Medicine. 1988;18:351–66.

3. Bers DM, Patton CW, Nuccitelli R. A practical guide to the preparation of Ca(2+) buffers. Methods Cell Biol. 2010;99:1–26.

4. Bers DM, Patton CW, Nuccitelli R. Chapter 1 - A Practical Guide to the Preparation of Ca2+ Buffers. In: Nuccitelli R, editor. Methods in Cell Biology. Academic Press; 1994. p. 3–29.

5. Hoff JH van't, Hoff JH. Études de dynamique chimique. F. Muller & Company; 1884.

6. Correction of proton and Ca association constants of EGTA for temperature and ionic strength. https://journals.physiology.org/doi/epdf/10.1152/ajpcell.1989.256.6.C1250. Accessed 10 Jun 2024.
